# A fast Monte Carlo cell-by-cell simulation for radiobiological effects in targeted radionuclide therapy using pre-calculated single-particle track standard DNA damage data

**DOI:** 10.3389/fnume.2023.1284558

**Published:** 2023-12-06

**Authors:** A. Lim, M. Andriotty, T. Yusufaly, G. Agasthya, B. Lee, C. Wang

**Affiliations:** ^1^ Nuclear & Radiological Engineering & Medical Physics Program, Georgia Institute of Technology, Atlanta, GA, United States; ^2^ Department of Radiology and Radiological Sciences, Johns Hopkins University, Baltimore, MD, United States; ^3^ Advanced Computing in Health Sciences Division, Oak Ridge National Laboratory, Oak Ridge, TN, United States; ^4^ Radiation Oncology Department, Stritch School of Medicine, Loyola University Chicago, Chicago, IL, United States

**Keywords:** Monte Carlo, radiation track structure, cell-by-cell radiobiological modeling, standard DNA damage data, DNA double-strand breaks

## Abstract

**Introduction:**

We developed a new method that drastically speeds up radiobiological Monte Carlo radiation-track-structure (MC-RTS) calculations on a cell-by-cell basis.

**Methods:**

The technique is based on random sampling and superposition of single-particle track (SPT) standard DNA damage (SDD) files from a “pre-calculated” data library, constructed using the RTS code TOPAS-nBio, with “time stamps” manually added to incorporate dose-rate effects. This time-stamped SDD file can then be input into MEDRAS, a mechanistic kinetic model that calculates various radiation-induced biological endpoints, such as DNA double-strand breaks (DSBs), misrepairs and chromosomal aberrations, and cell death. As a benchmark validation of the approach, we calculated the predicted energy-dependent DSB yield and the ratio of direct-to-total DNA damage, both of which agreed with published *in vitro* experimental data. We subsequently applied the method to perform a superfast cell-by-cell simulation of an experimental *in vitro* system consisting of neuroendocrine tumor cells uniformly incubated with ^177^Lu.

**Results and discussion:**

The results for residual DSBs, both at 24 and 48 h post-irradiation, are in line with the published literature values. Our work serves as a proof-of-concept demonstration of the feasibility of a cost-effective “*in silico* clonogenic cell survival assay” for the computational design and development of radiopharmaceuticals and novel radiotherapy treatments more generally.

## 1. Introduction

### 1.1. Dosimetric and radiobiological challenges of targeted radionuclide therapy (TRT)

**Figure 1:**
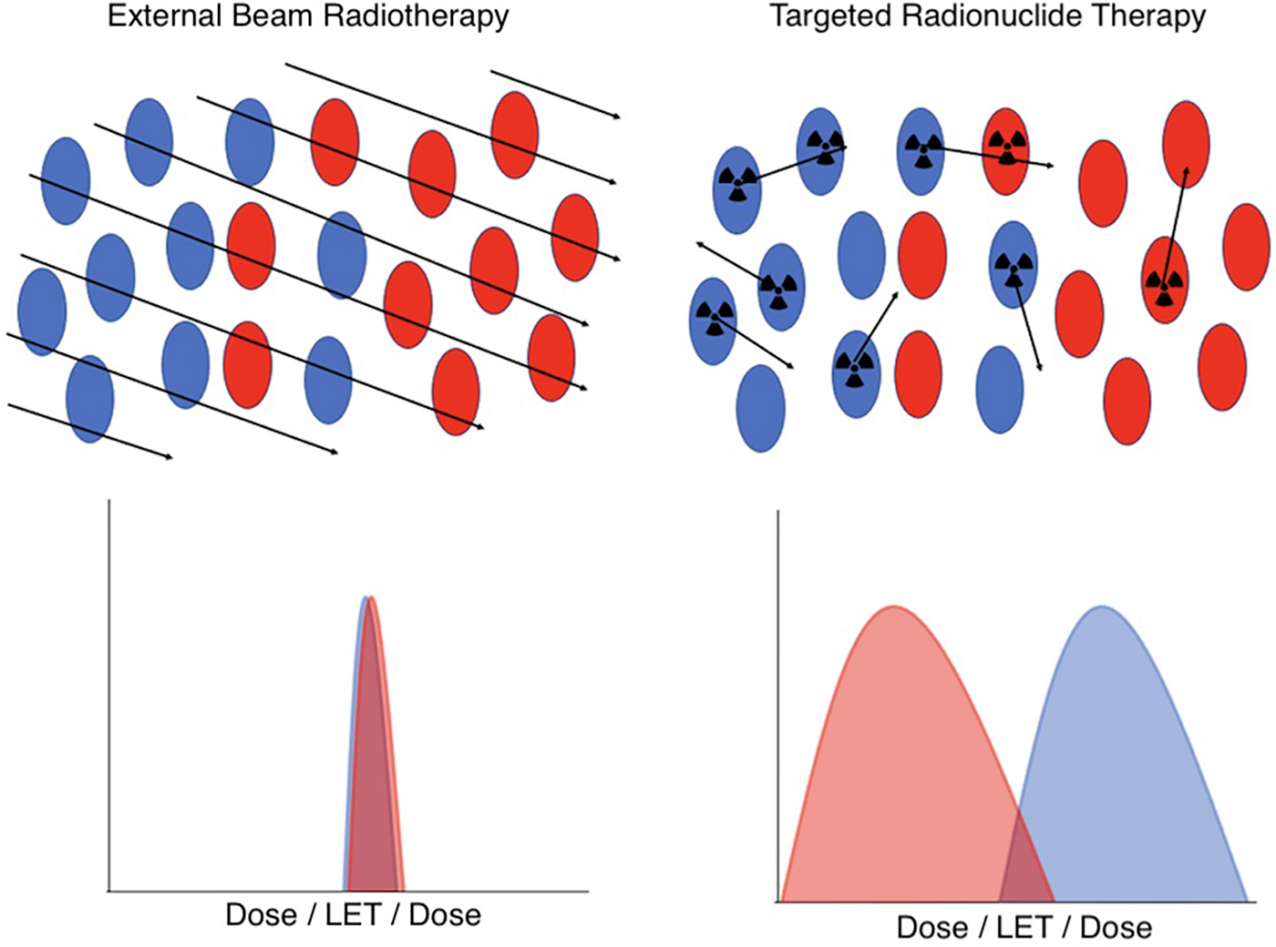
A schematic illustration of the key differences in dosimetry between EBRT (left) and TRT (right) at the cellular level. In EBRT, all cell types in a microscopic region of space have an essentially identical and uniform distribution of dose and radiation quality. However, in TRT, the cell-specific uptake of the radiopharmaceutical, coupled with the variable range of emitted radioactive tracks, results in heterogeneous and stochastic dose distributions and radiation qualities. Red indicates tumor cells, blue indicates normal cells.

Understanding therapeutic efficacy and normal tissue toxicity treatment planning for targeted radionuclide therapy targeted radionuclide therapy (TRT) presents special challenges compared to external beam radiotherapy (EBRT). These challenges arise from the unique characteristics of TRT dosimetry and its physiologically-mediated mechanism of radiation delivery, illustrated in Figure 1.

In a nutshell, the difference between TRT and EBRT comes down to the fact that in EBRT, all cells in a microscopic region of space are irradiated in a short timeframe with essentially identical dose and radiation quality, a simplicity that, in general, breaks down for TRT. The radiopharmaceuticals used in TRT include many classes of radionuclides that emit radiations of very different ranges and linear energy transfer (LET), including α particles, β particles, and Auger electrons. Moreover, the stochastic nature of radiopharmaceutical delivery at the microscale results in significant spatial and temporal variability in the dose distribution and biological effectiveness. In addition, the presence of radioactive daughters, which may or may not remain in the same compartments as their parents, further complicates predictive dosimetry modeling.

As a result of these dosimetric differences, the radiobiology of EBRT cannot be straightforwardly extrapolated to TRT. Especially in EBRT, the linear-quadratic (LQ) model (1) is widely used to assume biological equivalence and guide clinical decision-making. Traditional formulations of the LQ model, however, are designed for conventional low-LET photon or electron beams, sometimes with a few *ad hoc* extensions to account for additional complexities, such as dose rate or the high-LET of ion beams (2). However, due to the simplistic two-parameter nature of the LQ model, it is not clear how these traditional formulations should best be adapted to describe cell survival in the presence of the more complex patterns of cellular irradiation encountered in TRT (2, 3).

### 1.2. Advances in mechanistic modeling and simulation

As the previous discussions illustrate, true precision TRT dosimetry and treatment planning require a more principled approach to figuring out how to adapt the LQ models used in EBRT, a problem that remains widely open with ample opportunities for further research. In this regard, mechanistic radiobiological modeling, which explicitly simulates cellular and subcellular radiation chemistry, the production of complex DNA damage in the nucleus, and consequent cell death, can be an invaluable resource. In the last two decades, advances in these models have opened many new opportunities for *in silico* numerical “experiments”, which could potentially assist the community in understanding and solving outstanding issues in TRT radiobiology. The major breakthroughs can roughly be categorized into two groups: (1) Improvements in Monte Carlo-based radiation-track-structure (MC-RTS) codes (4–7), and (2) Advances in modeling the kinetics of DNA repair and cell survival.

Unlike traditional MC radiation transport codes, which simulate electron tracks in water only for energies above 1 keV, more recent MC-RTS codes are capable of simulating electron tracks for energies as low as a few eV. This extension is a crucial step toward reliable radiobiological calculations, as these low-energy electrons form nanometer-scale energy clusters and are frequently responsible for producing the most biologically consequential types of DNA damage, particularly complex double-strand breaks (DSBs). Of the numerous MC-RTS codes that exist, arguably the most well-known is TOPAS-nBio (7), an open-source package that simulates each individual radiation track traversing the cell nucleus one at a time. It includes detailed representations of cellular and subcellular biology and comprehensive tracking of various physical and chemical processes arising from ionizing radiation.

Concurrently, computer codes that model the kinetics of DNA damage repair, or misrepair, and the resulting cellular endpoints have rapidly developed over the past few years (8–10). The establishment of the Standard DNA Damage (SDD) data format (11) has facilitated model interoperability, allowing users to interface outputs from MC-RTS codes such as TOPAS-nBio into kinetic models of the DNA damage response, a prominent example being the recently developed Mechanistic DNA Repair and Survival Model (MEDRAS) (10). With the combined capabilities of TOPAS-nBio and MEDRAS, we are now at a point where we can simulate the kinetics of DSB repair and misrepair in the presence of arbitrarily complex cellular irradiation patterns, which in turn can be used to estimate more meaningful biological endpoints (e.g., chromosome aberrations and cell death).

### 1.3. Contributions to this work

The technical and methodological advances in radiobiological simulation described previously have opened many fruitful avenues for *in silico* single-cell exploration of the radiobiology of TRT and novel radiotherapy modalities more generally. However, major roadblocks remain that prevent broader usage of these tools. Namely, the simulation of subcellular radiochemistry and DNA damage remains computationally expensive. This bottleneck must be resolved if widespread adoption of *in silico* approaches for research and clinical applications is to be realized.

Our work here introduces a new method that circumvents computationally expensive MC-RTS calculations and makes cell-by-cell calculation of biological endpoints practical for arbitrarily complex irradiation scenarios. Prior work demonstrated the potential for cell-by-cell MC-RTS radiobiological modeling using pre-calculated libraries, and the presented work further accelerates efficiency while also accounting for radiochemistry (12). The method is based on randomly sampling and superimposing an ensemble of single-particle-track (SPT) SDD files from a “pre-calculated” SDD data library, and subsequently editing the data file with “time stamps” to incorporate dose-rate effects. This time-stamped SDD file can then be fed into MEDRAS to rapidly calculate biological endpoints.

In the next section, we explain how the pre-calculated single-electron-track (SET) SDD data library is constructed, and describe an application of our method to a low-dose-rate *in vitro* experiment of neuroendocrine tumor cells incubated with ^177^Lu-DOTATOC (13). In Section 3, we present our results. We first benchmark the method by showing that the SDD data agrees well with published *in vitro* measurements on the number of DSBs and the ratio of direct-to-indirect damage. We then show that the method is capable of reproducing observed time-dependent DSB yields in the *in vitro* ^177^Lu system, demonstrating that the pre-calculated SDD libraries can circumvent computationally demanding MC-RTS calculations and drastically speed up cell-by-cell simulation of radiation-induced effects without sacrificing accuracy. We conclude in Section 4, where we also discuss the limitations of the study and highlight areas for future work.

## 2. Methods

### 2.1. Using TOPAS-nBio to generate the single-electron-track SDD data library

The pre-calculated SET-SDD data library consists of many SET-SDD data files obtained from TOPAS-nBio runs for electrons of various energies. Each SET-SDD file consists of over 250,000 SET-SDD at each electron energy. The energies included in the data library span from 1 keV to 1 MeV, with intervals of 1 keV for energies between 1 keV and 20 keV, 5 keV for energies between 20 keV and 100 keV, 10 keV for energies between 100 keV and 500 keV, and 25 keV for energies between 500 keV and 1 MeV, resulting in a total of 96 energy-dependent SET-SDD files. Large amounts of SET-SDD data are necessary for the library to be adequately representative of the complete distribution of tracks and damage observed in a brute-force MC-RTS simulation.

**Figure 2:**
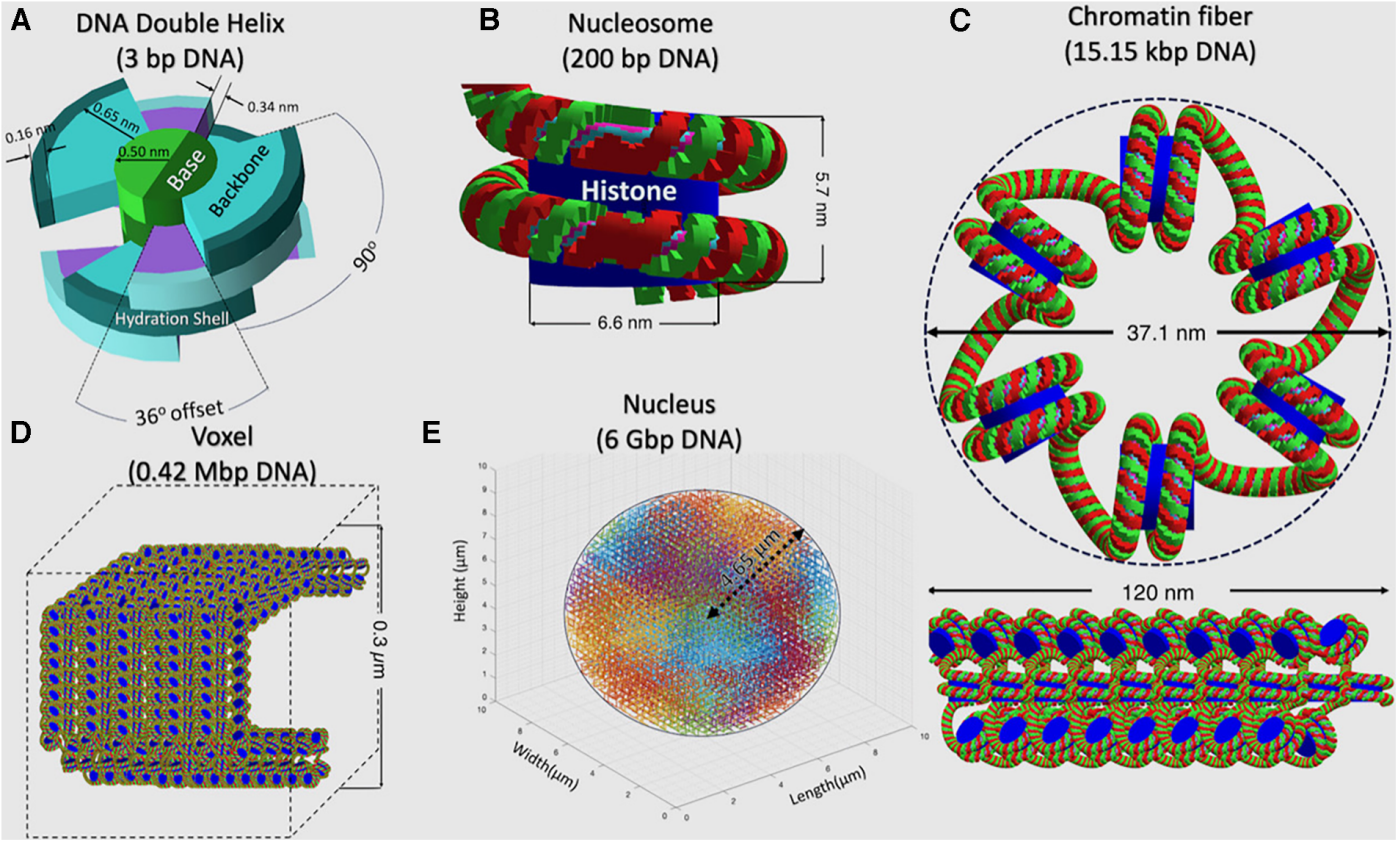
A detailed schematic, across a hierarchy of spatial scales, describing the built-in TOPAS-nBio G0/G1 cell nucleus model that was used to generate the SET-SDD data library. (**A**) DNA Double Helix, (**B**) Nucleosome, (**C**) Chromatin fiber, (**D**) Voxel filled with chromosome fiber, and (**E**) Cell nucleus.

In each TOPAS-nBio run, the cell nucleus is modeled according to a G0/G1 human fibroblast, shown in Figure 2, which is the default option available in TOPAS-nBio. The nucleus is a sphere with a diameter of 9.3 μm and 46 chromosomes (Figure 2E). Chromatin fibers are folded according to a continuous 3D Hilbert space-filling curve to form chromatin fiber loops and fill a voxel (Figure 2D). Each chromatin fiber has a radius of 37.1 nm and a length of 120 nm and consists of 51 nucleosomes and 15,150 base pairs (bps) of DNA (Figure 2C), resulting in 14,328 voxels and 6.08 Gbp DNA for the whole nucleus. The nucleosome consists of a histone protein complex, modeled as a cylinder with a diameter of 6.6 nm and a height of 5.7 nm (Figure 2B). The DNA double helix is modeled as a half-cylinder structure (Figure 2A), where a pair of bases is linked to the next following the DNA double helix structure, with a thickness of 0.34 nm. The inner half-cylinder base volume has a radius of 0.5 nm, and the outer quarter-cylinder backbone volume has a radius of 1.15 nm. Finally a hydration shell of thickness 0.16 nm is added as an external layer.

Each electron's history starts with a randomly selected position on the cell nucleus surface. The electron's initial traveling direction is randomly selected in an isotropic manner, inwardly toward the nucleus. A single electron track data file contains information on energy transfer, chemical species generated, and track location within the cell nucleus. The physics module recommended for use in water radiolysis, TsEmDNAPhysics, is used for physical stage simulation, with a direct strand break being formed when at least 17.5 eV of energy is deposited within the DNA backbone volume and neighboring hydration shell. The TsEmDNAChemistry module is then used to simulate the chemical interactions in the pre-chemical and chemical stages, with the length of the chemical stage simulation set to 1 ns. While all the radiolysis products are included in the simulation, only interactions between hydroxyl radicals (^•^OH) and the DNA backbone are assumed to induce indirect strand breaks. A probability of 0.4 is assumed for the ^•^OH-induced indirect strand break, based on previously reported values (14). A DSB is formed when two single-strand breaks (SSBs) occur on opposite sides of the DNA within a distance of 10 bps.

This resulting DNA damage data, including base damage (BD), SSBs, and DSBs, is recorded in SDD format, and can be directly used as input into a DNA damage response model to compute repairs and misrepairs, along with subsequent chromosome aberrations and cell death, as we will describe next.

### 2.2. Cell-by-cell simulation using the pre-calculated SET-SDD data library: *in vitro* Lu-177 as a demonstrative example

**Figure 3:**
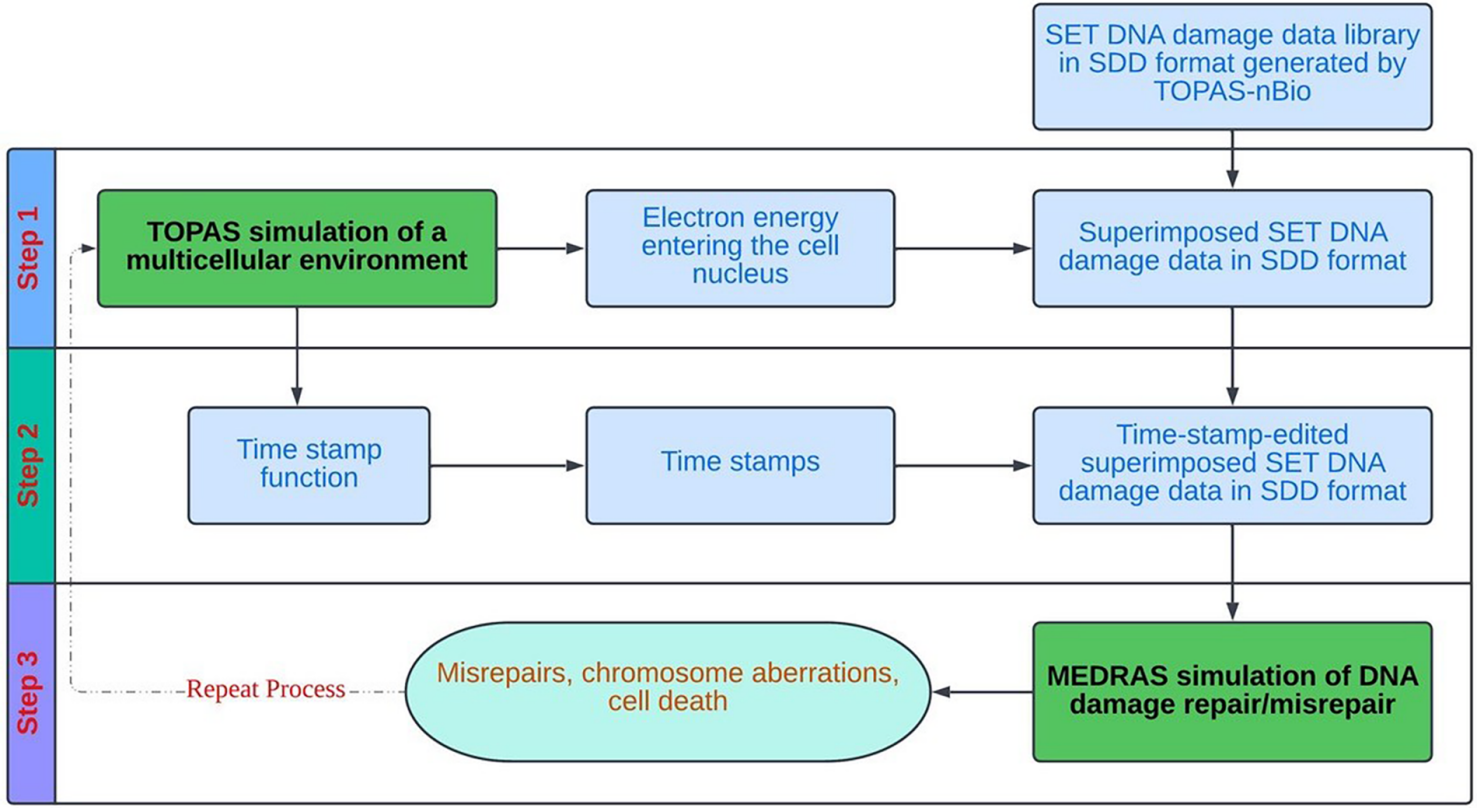
Overall process flow diagram for the cell-by-cell radiobiological simulation, based on random sampling and superposition of SET SDD files from a pre-calculated data library.

Figure 3 provides a schematic overview of the overall flow of the cell-by-cell simulation using the precalculated data library. As shown, the simulation works on one cell at a time, and each cell goes through three steps: (1) sampling and superposition of SET-SDD data from the pre-calculated library to simulate a total delivered dose; (2) editing the superimposed SDD data file obtained from step (1) with proper “time stamps” based on a specified dose rate; and (3) taking the time-stamped SDD data as the input into the MEDRAS code to calculate biological end points as a function of time, including residual DSBs, misrepairs, and chromosomal aberrations. The process is repeated for many cell replicates until the sample statistics have converged below the desired error tolerance. In the following paragraphs, we will describe the details of each step in the process more explicitly, in the context of the concrete example of an *in vitro* Lu-177 experiment.

#### 2.2.1. Step 1

**Figure 4:**
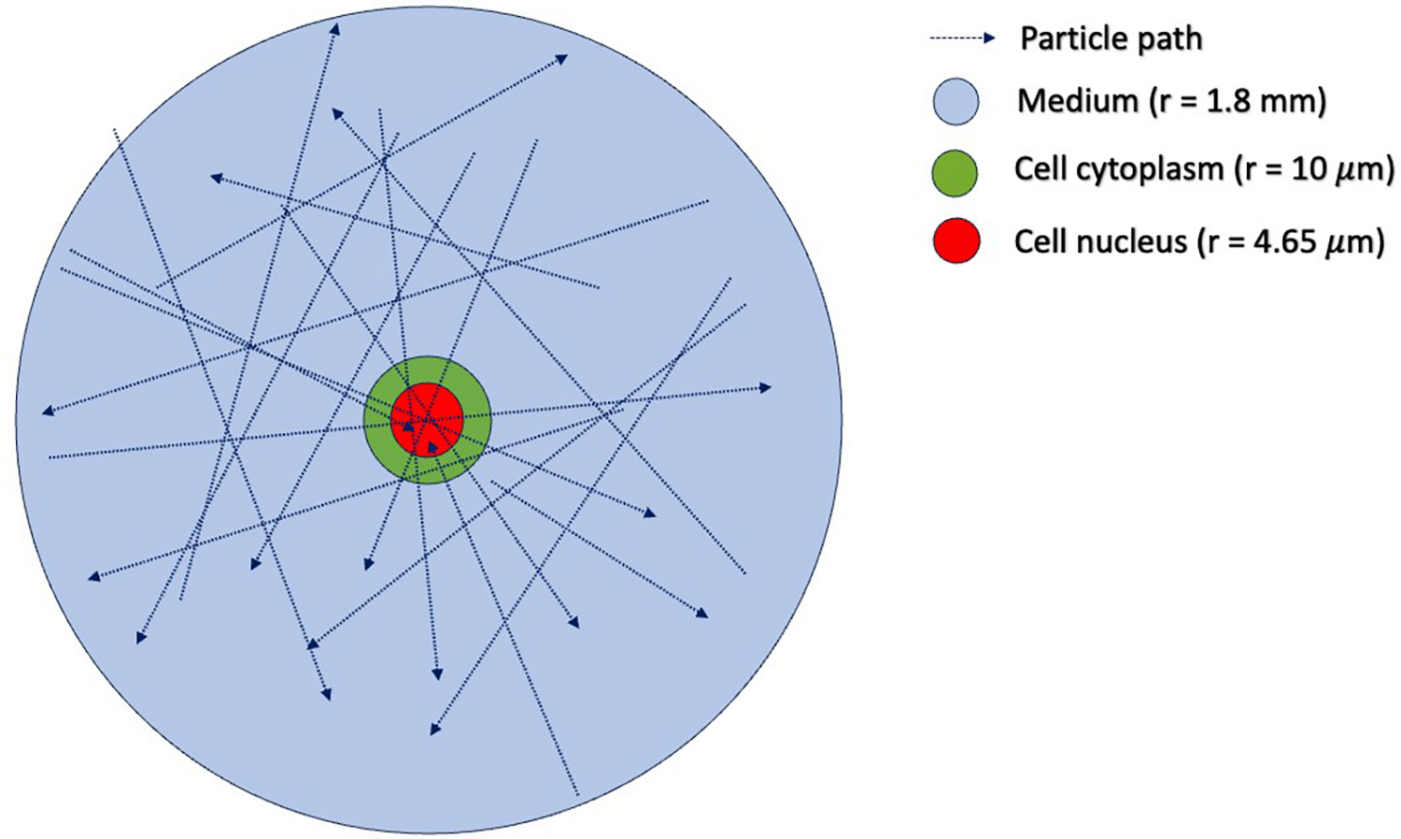
Simulation geometry in TOPAS-MC used to estimate the distribution of incident electron tracks for cells irradiated with ^177^Lu. The outer blue sphere has a radius of 1.8 mm, corresponding to the range of the maximum energy of the ^177^Lu beta particle emission (498 keV). The green sphere represents the cell cytoplasm, with a radius of 10 μm. The red sphere in the center represents the cell nucleus, with a radius of 4.65 μm. The initial positions of the beta-emitting ^177^Lu particles are uniformly distributed in the volume outside the cell nucleus.

In step (1), one must first determine the sampling distribution of electron energies that are incident on the cell nucleus. To do this, we use TOPAS (15) to simulate an ensemble of electrons emitted from Lu-177, traveling and interacting under the *in vitro* condition. The geometry of the TOPAS simulation is shown in Figure 4, which includes a water sphere (representing the medium) with an outer radius of 1.8 mm and a target cell at the center. The 1.8 mm radius corresponds to the range of the maximum energy of the beta particles (498 keV) emitted from ^177^Lu. The target cell is modeled as two concentric water spheres with radii of 4.65 μm and 10 μm, respectively. The electron initial positions are modeled to be uniformly distributed in the cell's medium and cytoplasm, with isotropic directions of travel, as previously reported (16). The initial energy of each electron is determined by sampling the Lu-177 beta spectrum obtained from RADAR (17). We ignore the dose contribution from the Auger electrons, as their yields are negligible and their ranges are too short to reach the cell nucleus. From this ensemble, we tally both the electron's energy when it enters the cell nucleus and the amount of energy deposited in the nucleus by the electron. This observed distribution can then be used to convert a given ^177^Lu activity concentration (in MBq/ml) into the desired absorbed dose rate for the cell nucleus under *in vitro* irradiation. To construct an SDD file for a cell nucleus at a given total absorbed dose, we randomly sample from the precalculated SET-SDD library until the specific energy to the cell nucleus matches the desired dose.

Table 1 shows the TOPAS-estimated conversion from the cumulative activity concentration of Lu-177 to the absorbed dose to the cell nucleus during the irradiation period. It should be noted that the absorbed dose values shown in Table 1 are one-half that of the TOPAS results. This is because in the TOPAS simulation (Figure 4), the cell is fully engulfed in the medium containing beta-emitting Lu-177. In actual *in vitro* experiments, however, the cell attaches itself to the bottom of the flask and is only exposed to half of the beta particles emitted from Lu-177.

**Table 1:** Initial dose rate and cumulative doses to the cell nucleus, determined through TOPAS simulations of *in vitro* irradiation with 10 MBq/ml of ^177^Lu.

Initial dose rate	0.67 Gy/h
Irradiation time (h)	Cumulative dose to cell nucleus (Gy)
24	15.2
48	28.9
72	41.2

#### 2.2.2. Step 2

In step (2), we edit the superimposed SDD file with a time stamp for each electron track, including information on both the time and location of the DNA damage within the cell nucleus. Specifically, the time stamp refers to the time difference between two consecutive electrons traversing the cell nucleus. To obtain the time stamp for the first electron, we assume that the count rate of electron tracks traversing the cell nucleus is proportional to the dose rate reported in Table 1. That is, ${\dot{N}}_{o}=k{\dot{D}}_{o}$, where ${\dot{N}}_{o}$ is the initial count rate of electron tracks traversing the cell nucleus, ${\dot{D}}_{o}$ is the initial dose rate, and *k* is a constant. The time stamp $TS_{1}$ of the first electron track is then:

$$TS_{1}=\frac{1}{{\dot{N}}_{o}}$$

To account for the fact that the dose rate decreases in proportion to the decay of Lu-177, the time stamps for the subsequent electron tracks at time *t* are calculated as:

$$TS_{n}=\frac{1}{\dot{N}(t)}=\frac{TS_{n-1}}{e^{-\lambda \cdot TS_{n-1}}}$$

#### 2.2.3. Step 3

In step (3), the time-stamped SDD file is fed as input to the MEDRAS code (10) to simulate how the cell processes the DNA damage. The MEDRAS code considers three DSB repair pathways: “fast” Non-Homologous End Joining (NHEJ), “slow” Homologous Recombination (HR), and “very slow” Microhomology-Mediated End Joining. There are 11 parameters to model repair kinetics, including the repair rate coefficients and repair and misrepair probabilities for the three pathways. NHEJ is the major DNA repair pathway for G0/G1 human fibroblasts of the type built-in to TOPAS and used to construct the SET SDD library, and as a result, we neglected HR and MMEJ for this simulation study. The calculated outputs of MEDRAS that we analyze in this work include residual DSBs, misrepairs, and lethal chromosome aberrations.

## 3. Results and discussion

### 3.1. Benchmark validation of the SET-SDD library data

**Figure 5:**
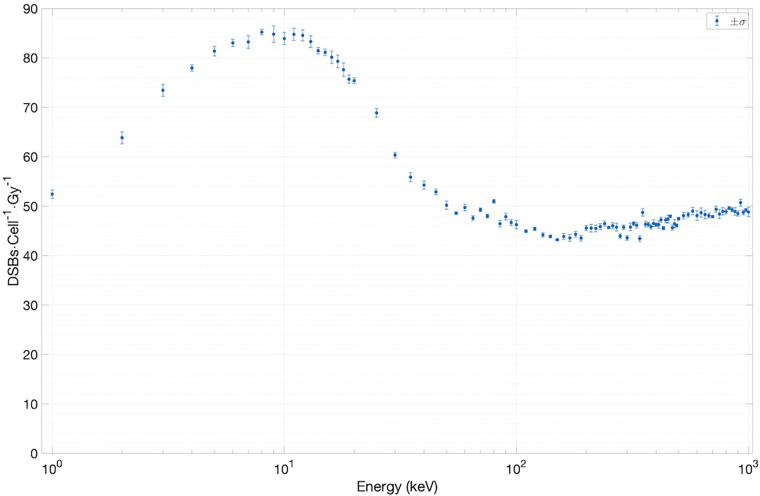
Results for yield of DSBs cell^−1^ Gy^−1^ versus electron energy.

**Figure 6:**
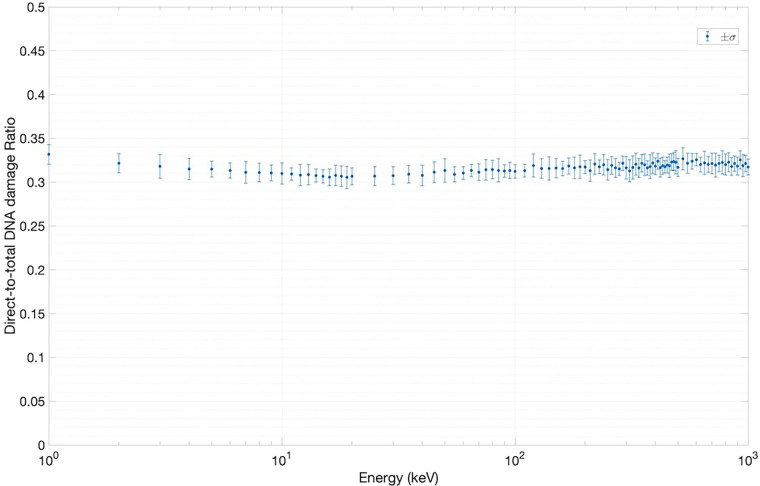
Results for direct-to-total DNA damage ratio versus electron energy.

We have used two experimentally measurable quantities to validate the SET-SDD library data obtained via the TOPAS-nBio simulation runs described in Section 2.1: the yield of DSBs/cell/Gy (Figure 5) and the direct-to-total DNA damage ratio (Figure 6).

To estimate the yield of DSBs/cell/Gy as a function of electron energy, we have extracted the total number of DSBs (∼10,000) from an ensemble of SET-SDD data available for each electron energy in the library. The statistical error associated with each data point was observed to be approximately 1%. As shown in Figure 5, the yield settles between 45 and 50 DSBs/cell/Gy for electrons with energies greater than ∼40 keV. Below 40 keV, the value increases steadily, reaching a peak of 80 DSBs/cell/Gy at ∼10 keV, in reasonable agreement with previously published experimental results (18, 19).

Figure 6 shows the results of the direct-to-total DNA damage ratio versus electron energy. As in Figure 5, each data point in Figure 6 was extracted using an ensemble of SET-SDD data available for each electron energy in the library, with a statistical error associated with each data point of approximately 3%. As shown, the value of the direct-to-total DNA damage ratio settled to approximately 0.3 over the entire electron energy range, once again consistent with previously published experimental data (20).

### 3.2. *In vitro* Lu-177 simulation

**Figure 7:**
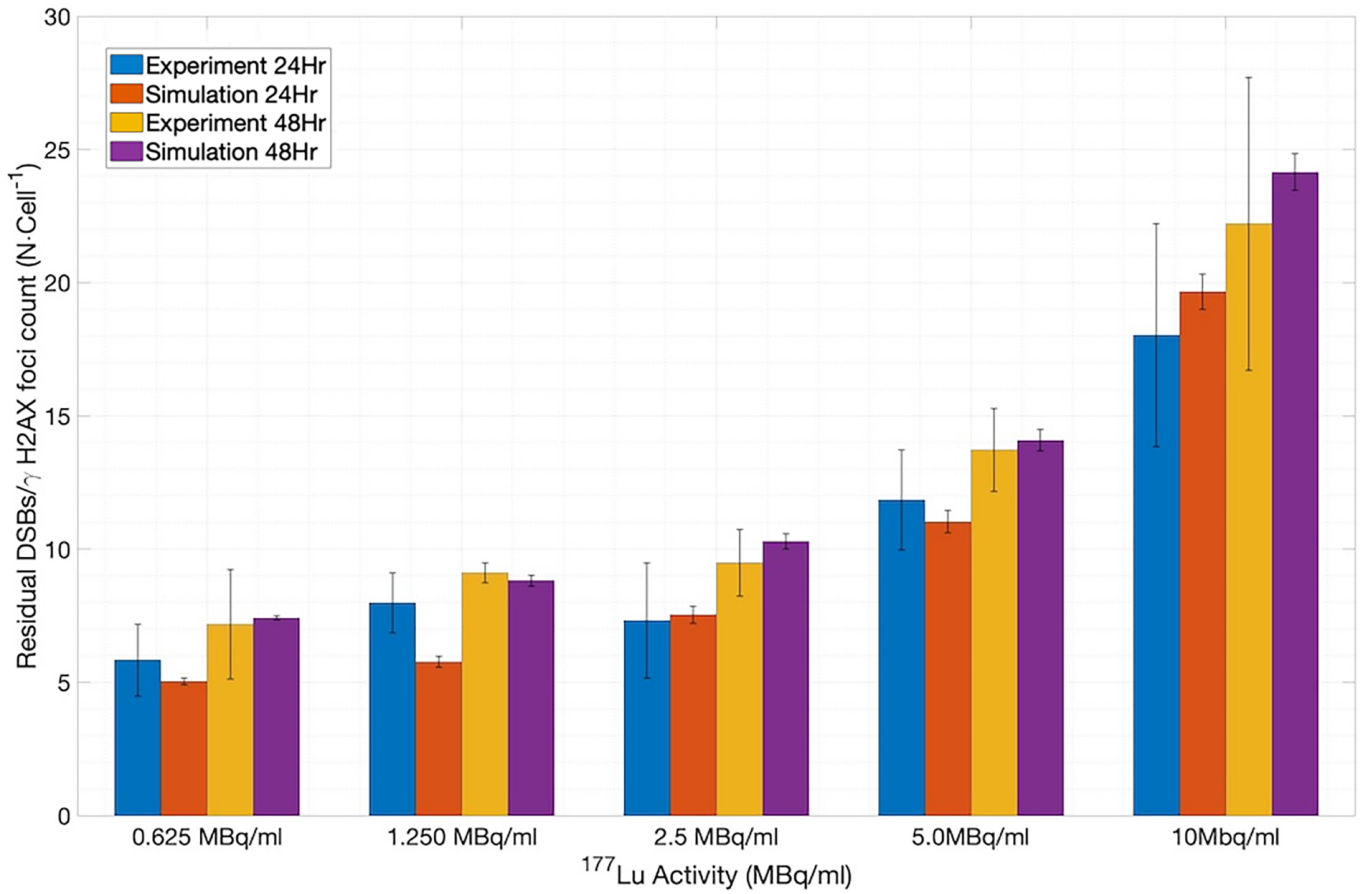
Comparison between the number of residual DSBs cell^−1^ output from MEDRAS and the number of γH2AX foci cell^−1^ obtained in the *in vitro* experiments.

To assess the application of the new SDD library-based simulations to the ^177^Lu system, we extracted the published *in-vitro* experimental data on the number of γH2AX foci per cell measured at 24 and 48 h of incubation for various concentrations of ^177^Lu-DOTATOC (5) and compared them with the number of residual DSBs output from MEDRAS. As shown in Figure 7 (constructed using 1,200 cells), the simulations and experiments agree well (12). We note that the simulated results included a baseline rate of 4.8 DSBs cell^−1^ on top of the rate calculated by MEDRAS to account for the background number of γH2AX foci per cell observed experimentally.

**Figure 8:**
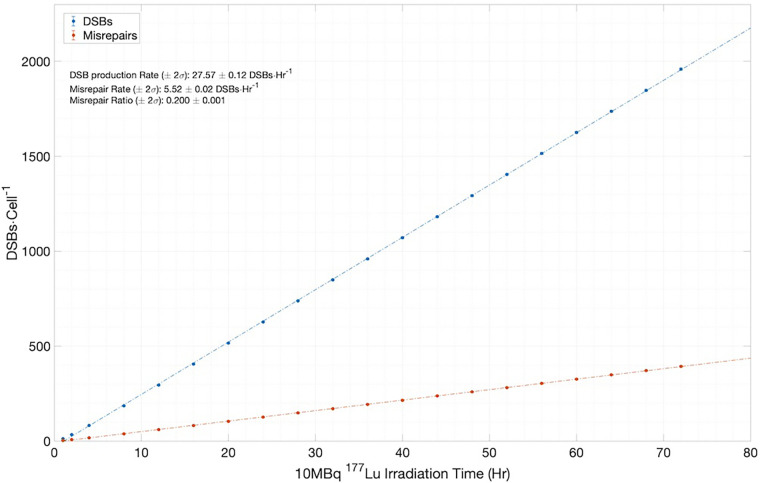
Simulation results for the number of DSBs and misrepairs, output from MEDRAS for cells incubated with 10 MBq/ml of ^177^Lu over a 72 h period.

However, to fully appreciate the results of Figure 7, it is instructive and illuminating to closely examine the time-dependent results obtained from MEDRAS, as shown in Figure 8. We would like to draw attention to two findings here: (1) the production rate of DSBs is relatively constant at 27.6 DSBs/cell/h throughout the incubation period, and (2) the number of DSBs produced over the incubation, which was found to be much greater than the number of residual DSBs measured at the two time points, i.e., 24 and 48 h. These two findings provided a more detailed description of the results than shown in Figure 7. Because the rate of DSB production was low relative to the rate of repair via NHEJ, the overwhelming majority (>98%) of DSBs during the irradiation period were repaired or misrepaired during the incubation period, resulting in only a small number of residual DSBs per cell after 48 h. This is further demonstrated in Figure 8, which shows additional results on the number of DSBs produced and misrepairs. Our results of the number of residual DSBs shown in Figure 7 (specifically for 2.5 MBq/ml) are much lower than those of a recent paper by Rumiantcev et al. (21), which also used TOPAS-nBio and MEDRAS. The difference may be caused by the time stamps we added in the MEDRAS simulation to reflect the dose-rate effect. In contrast, the paper assumed acute dose delivery.

### 3.3. Computational efficiency of the SDD library-based simulation method

To evaluate the computational efficiency of the new SDD library-based simulation method, we compared the computation time using the new method to that using the brute-force track-by-track TOPAS-nBio simulation for a single cell irradiated with 41 Gy of dose, corresponding to 10 MBq of 72 h irradiation with Lu-177. The computation times for the new method and the track-by-track TOPAS-nBio simulation were 31.8 s and 2.52 days, respectively, on an Apple M1 Max laptop. In other words, our new method sped up the simulation by approximately 4 orders of magnitude. In fact, using Georgia Tech's high-performance computer, we were able to complete the simulation of more than 1,200 cells irradiated with 41 Gy of Lu-177 beta particles in a few minutes.

## 4. Conclusions and future work

In conclusion, we have constructed an SET-SDD data library for electrons of various energies using TOPAS-nBio and used it to enable superfast calculations via a novel superposition algorithm based on randomly sampling pre-calculated SET-SDD data from the library. The new method drastically speeds up MC-RTS simulations of DNA damage, allowing rapid evaluation of how cells respond to radiation-induced DNA damage on a cell-by-cell basis. At the same time, it is capable of reproducing experimental observations with an accuracy comparable to full MC-RTS calculations.

While this initial study developed the method in the context of low-LET beta particles, the generalization to high-LET alpha particles is a natural next step, particularly in light of the growing popularity of alpha-emitting TRT for aggressive and metastatic disease. This generalization should, in principle, be straightforward. However, given the vastly increased computational complexity of alpha particle simulations, future work might consider using a GPU-accelerated MC-RTS platform, such as gMicroMC (22), for more efficient library generation. In addition, special precautions need to be taken when adapting the new method to short-range particles. For these irradiation scenarios, the SPT-SDD data generated by a given simulation becomes highly dependent on the geometric configuration of the cell nucleus model and on the spatial and directional distributions of the source particles. Framed another way, it becomes imperative to go beyond the “continuum approximation” that has been used for long-range beta particle dosimetry in this work. For shorter-range alpha particles, it is no longer sufficient to represent the activity concentration as a uniform continuous entity, and the explicit discrete cellular and subcellular localization must be accounted for. In this regard, it is worth noting that for dosimetry models and algorithms to be useful for alpha-emitters, whole-organ or tumor estimates of the total activity and absorbed dose will need to be supplemented with small-scale models of the microscopic sub-resolution distribution. These “sub-grid” rules typically must be inferred indirectly from alpha camera autoradiographic imaging in surrogate preclinical and/or animal systems (23).

More generally, beyond TRT for a reference cell nucleus, the approach can be adapted to develop libraries for other forms of ionizing radiation (e.g., x-ray/gamma photons, protons, alpha particles) and for different cell types (as represented by different chromatin structures and radiochemistry). In regards to the latter generalization, it is particularly worth mentioning the emerging recognition that the dynamic chromatin architecture throughout the cell cycle plays an important role in regulating radiosensitivity, in addition to the dynamics of DNA repair pathways (24). This dynamic architecture is determined by the complex interplay of many different factors, in particular the histone electric charge and its influence on electron transport in the nucleosome (25) as well as the Hi-C data from different cell types (26).

In summary, this study should pave the way for more widespread applications of *in silico* radiobiology for the discovery and optimization of novel radiotherapy modalities and dose delivery schemes.

## Data Availability

The raw data supporting the conclusions of this article will be made available by the authors, without undue reservation.

## References

[1] Bentzen SM Joiner MC. The linear-quadratic approach in clinical practices. In: Joiner M van der Kogel A, editors. Basic clinical radiobiology. 4th ed. Baca Raton, FL: CRC Press (2009). p. 120–34.

[2] Wang C. The progress of radiobiological models in modern radiotherapy with emphasis on the uncertainty issue. Mutat Res. (2010) 704:175–81. 10.1016/j.mrrev.2010.02.001 20178860

[3] Sgouros G Roeske J McDevitt M Palm S Allen B Fisher D MIRD pamphlet no. 22—radiobiology and dosimetry of alpha emitters for targeted radionuclide therapy. J Nucl Med. (2010) 51(2):311–28. 10.2967/jnumed.108.058651 20080889 PMC5680544

[4] Kreipl M Friedland W Paretzke H. Time- and space-resolved Monte Carlo study of water radiolysis for photon, electron and ion irradiation. Radia Environ Biophys. (2009) 48:11–20. 10.1007/s00411-008-0194-8 18949480

[5] Plante I Ponomarev A Patel Z Slaba T Hada M. RITCARD: radiation-induced tracks, chromosome aberrations, repair and damage. Radiat Res. (2019) 192:282–98. 10.1667/RR15250.1 31295089

[6] Bernal M Bordage M Brown J Davídková M Delage E El Bitar Z Track structure modeling in liquid water: a review of the Geant4-DNA very low energy extension of the Geant4 Monte Carlo simulation toolkit. Phys Med. (2015) 31(8):861–74. 10.1016/j.ejmp.2015.10.087 26653251

[7] Schuemann J McNamara AL Ramos-Méndez J Perl J Held KD Paganetti H TOPAS-nBio: an extension to the TOPAS simulation toolkit for cellular and subcellular radiobiology. Radiat Res. (2019) 191:125–38. 10.1667/RR15226.1 30609382 PMC6377808

[8] Taleei R Nikjoo H. Biochemical DSB-repair model for mammalian cells in G1 and early S phases of cell cycle. Mutat Res. (2013) 756:206–12. 10.1016/j.mrgentox.2013.06.004 23792210

[9] Ponomarev A George K Cucinotta F. Computational model of chromosome aberration yield induced by high- and loe-LET radiation exposures. Radiat Res. (2012) 177:727–37. 10.1667/RR2659.1 22490019

[10] McMahon S Prise K. A mechanistic DNA repair and survival model (MEDRAS): applications to intrinsic radiosensitivity, relative biological effectiveness and dose-rate. Front Oncol. (2021) 11:1–18. 10.3389/fonc.2021.689112 PMC8276175 34268120

[11] Schuemann J McNamara AL Warmenhoven JW Henthorn NT Kirkby KJ Merchant MJ A new standard DNA damage (SDD) data format. Radiat Res. (2019) 191:76–92. 10.1667/RR15209.1 30407901 PMC6407706

[12] Lee B Wang C. A cell-by-cell Monte Carlo simulation for assessing radiation-induced DNA double strand breaks. Phys Med. (2019) 62:140–51. 10.1016/j.ejmp.2019.05.006 31153394

[13] Graf F Fahrer J Maus S Morgenstern A Bruchertseifer F Venkatachalam S DNA double strand breaks as predictor of efficiency of the alpha-particle emitter Ac-225 and the electron emitter Lu-177 for somatostatin receptor targeted radiotherapy. PLoS One. (2014) 9(2):1–10. 10.1371/journal.pone.0088239 PMC3917860 24516620

[14] Zhu H McNamara AL McMahon SJ Ramos-Mendez J Henthorn NT Faddegon B Cellular response to proton irradiation: a simulation study with TOPAS-nBio. Radiat Res. (2020) 194:9–21. 10.1667/RR15531.1 32401689 PMC7484135

[15] Perl J Shin J Schümann J Faddegon B Paganetti H. TOPAS: an innovative proton Monte Carlo platform for research and clinical applications. Med Phys. (2012) 39(11):6818–37. 10.1118/1.4758060 23127075 PMC3493036

[16] Tamborino G De Saint-Hubert M Struelens L Seoane DC Ruigrok EAM Aerts A Cellular dosimetry of ^177^[Lu]Lu-DOTA-[Tyr3]octreotate radionuclide therapy: the impact of modeling assumptions on the correlation with in vitro cytotoxicity. EJNMMI Phys. (2020) 7:8. 10.1186/s40658-020-0276-5 32040783 PMC7010903

[17] Radiation Dose Assessment Resource (RADAR). Available at: https://www.doseinfo-radar.com/RADARDecay.html

[18] Rothkamm K Krüger I Thompson L Löbrich M. Pathways of DNA double-strand break repair during the mammalian cell cycle. Mol Cell Biol. (2003) 23:5706–15. 10.1128/MCB.23.16.5706-5715.2003 12897142 PMC166351

[19] Ward J. DNA damage produced by ionizing radiation in mammalian cells: identities, mechanisms of formation and repairability. Prog Nuclei Acid Res Mol Biol. (1988) 35:95–125. 10.1016/S0079-6603(08)60611-X 3065826

[20] Ward J. Biochemistry of DNA lesions. Radiat Res. (1985) 104(2II):103–11. 10.2307/3576637 3867077

[21] Rumiantcev M Li WB Lindner S Liubchenko G Resch S Bartenstein P Estimation of relative biological effectiveness of ^225^Ac compared to ^177^Lu during [^225^Ac-PSMA and [^177^Lu]Lu-PSMA radiopharmaceutical therapy usig TOPAS/TOPAS-nBio/MEDRAS. EJNMMI Phys. (2023) 10:53. 10.1186/s40658-023-00567-2 37695374 PMC10495309

[22] Tsai MY Tian Z Qin N Yan C Lai Y Hung SH A new open-source GPU-based microscopic Monte Carlo simulation tool for the calculations of DNA damages caused by ionizing radiation—Part I: core algorithm and validation. Med Phys. (2020) 47(4):1958–70. 10.1002/mp.14037 31971258

[23] Sgouros G Bolch W Chiti A Dewaraja Y Emfietzoglou D Hobbs R ICRU 96 dosimetry-guided radiopharmaceutical therapy. J ICRU. (2021) 21(1):1–212. 10.1177/14736691211060117

[24] Sitmukhambetov S Dinh B Lai Y Banigan EJ Pan Z Jia X Development and implementation of a metaphase DNA model for ionizing radiation induced DNA damage calculation. Phys. Med. Biol. (2023) 68:014001. 10.1088/1361-6560/aca5ea PMC9969557 36533598

[25] Fenley AT Adams DA Onufriev AV. Charge state of the globular histone core controls stability of the nucleosome. Biophys. J. (2010) 99(5):1577–85. 10.1016/j.bpj.2010.06.046 20816070 PMC2931741

[26] Sanders JT Freeman TF Xu Y Golloshi R Stallard MA Hill AM Radiation-induced DNA damage and repair effects on 3D genome organization. Nat Commun. (2020) 11:6178. 10.1038/s41467-020-20047-w 33268790 PMC7710719

